# Lactate dehydrogenase A exhibits sorbitol/fructose-related catalytic activity and contributes to polyol pathway metabolism in tumor cells

**DOI:** 10.3389/fendo.2026.1848426

**Published:** 2026-07-20

**Authors:** Siting Feng, Zhiyuan Hu, Hanbing Tong, Hong Zhang

**Affiliations:** 1 First Affiliated Hospital, Dalian Medical University, Dalian, Liaoning, China; 2 Central Hospital of Dalian University of Technology, Faculty of Medicine, Dalian University of Technology, Dalian, Liaoning, China

**Keywords:** cancer metabolism, hypoxia, lactate dehydrogenase A, polyol pathway, sorbitol dehydrogenase

## Abstract

**Introduction:**

Cancer cells undergo metabolic reprogramming to support survival under stress conditions. Although the polyol pathway has been extensively studied in diabetes, its role in tumor metabolism remains incompletely understood. This study investigated whether lactate dehydrogenase A (LDHA), a key glycolytic enzyme, contributes to hypoxia-associated sorbitol/fructose metabolism in U87MG tumor cells with relatively low sorbitol dehydrogenase (SDH) expression.

**Methods:**

Intracellular metabolites in normoxic and hypoxic U87MG cells were analyzed by mass spectrometry. Recombinant SDH and LDHA were expressed in Escherichia coli, purified, and subjected to comparative kinetic analysis at 25°C and 37°C. LDHA knockdown and overexpression were performed to evaluate intracellular sorbitol and fructose levels, ROS accumulation, and cell viability under hypoxia.

**Results:**

Metabolomic analysis showed marked fructose accumulation in hypoxic U87MG cells. Both recombinant SDH and LDHA catalyzed sorbitol oxidation and fructose reduction in vitro, and LDHA showed measurable catalytic turnover at 37°C. Cellular analysis showed that LDHA was readily detectable and increased under hypoxia, whereas SDH remained relatively low. LDHA knockdown reduced intracellular fructose levels, increased sorbitol accumulation, elevated ROS levels, and decreased cell viability under hypoxia, whereas LDHA overexpression showed the opposite trend.

**Discussion:**

These findings provide biochemical and cellular evidence that LDHA contributes to hypoxia-associated sorbitol/fructose metabolism and links this metabolic activity to redox balance and cell viability in U87MG cells.

## Introduction

Cancer is one of the most intensively studied and costly diseases worldwide. As early as the 1920s, the German biochemist Otto Warburg described a major metabolic feature of tumor cells, namely a high rate of glycolysis. Warburg proposed that mitochondrial respiratory dysfunction is an initiating event in tumorigenesis, forcing cells to rely on an alternative metabolic mode, aerobic glycolysis, to sustain survival and satisfy the demand for macromolecular biosynthesis (1). Although glycolysis is less efficient than oxidative phosphorylation in terms of ATP production, it is indispensable for rapidly proliferating tumor cells because it not only supplies energy but also provides intermediates required for the synthesis of biological macromolecules, thereby conferring a growth advantage (2).

Under normal conditions, glucose entering mammalian cells is rapidly phosphorylated to glucose-6-phosphate by hexokinase. However, when glucose availability is increased, the polyol pathway can be activated. This pathway consists of two sequential reactions catalyzed by aldose reductase and sorbitol dehydrogenase. Aldose reductase is a key enzyme in the polyol pathway and plays an important role in the development of several diabetic complications (3). In the first step of this pathway, aldose reductase uses nicotinamide adenine dinucleotide phosphate (NADPH) as a cofactor to reduce glucose to sorbitol (4, 5). When intracellular glucose levels are elevated, sorbitol can accumulate and subsequently be oxidized to fructose by sorbitol dehydrogenase. Excessive accumulation of sorbitol may increase osmotic stress and has been implicated in the pathogenesis of diabetic complications such as cataracts, neuropathy, and nephropathy (6).

Sorbitol dehydrogenase (SDH), the second enzyme of the polyol pathway, utilizes nicotinamide adenine dinucleotide (NAD^+^) as a cofactor and is a zinc-containing enzyme involved in polyol metabolism (7. 8). SDH catalyzes the conversion of sorbitol to fructose and is particularly important in tissues such as the liver, lens, retina, kidney, placenta, and sperm cells (9). Deficiency or low activity of SDH, especially under hyperglycemic conditions, can lead to intracellular sorbitol accumulation, resulting in osmotic swelling, altered membrane permeability, oxidative stress, and eventually tissue injury (10–12).

It has been reported that sorbitol dehydrogenase is expressed at low levels in many cell types, including some tumor cells (13). However, tumor cells exhibit profound metabolic reprogramming and remain capable of maintaining active carbon flux under stressful conditions such as hypoxia. In our study, metabolomic analysis showed marked accumulation of fructose in hypoxic U87MG cells, suggesting that sorbitol-to-fructose conversion may still occur even when SDH expression is limited. This observation led us to hypothesize that another enzyme with catalytic properties similar to those of SDH might participate in this process. Lactate dehydrogenase (LDH), one of the key enzymes in glycolysis, catalyzes the reversible conversion of pyruvate to lactate, coupled to the interconversion of NADH and NAD^+^. LDH is composed of two major subunits, LDH-A and LDH-B, which assemble into five isoenzymes (LDH1, LDH2, LDH3, LDH4, and LDH5). LDH-A, encoded by the LDHA gene, is the predominant form in skeletal muscle and has a relatively high affinity for pyruvate (14).

Because enzymatic promiscuity has been described for a number of metabolic enzymes, it is important to compare the catalytic activities and kinetic properties of SDH and LDHA directly. In the present study, we examined hypoxia-associated sorbitol/fructose metabolism in U87MG tumor cells and compared the sorbitol/fructose-related catalytic activities of recombinant SDH and LDHA *in vitro*. We further performed LDHA knockdown and overexpression experiments to determine whether LDHA affects intracellular sorbitol/fructose balance, ROS accumulation, and cell viability under hypoxic conditions. Our findings provide biochemical and cellular evidence that LDHA possesses measurable sorbitol/fructose-related catalytic activity and contributes to hypoxia-associated sorbitol/fructose metabolism in U87MG cells with relatively low SDH expression.

## Materials and methods

### Materials

Glucose-6-phosphate, glucose-6-phosphate dehydrogenase, fructose, sorbitol, protein assay reagent, NADH, and NAD^+^ were obtained from Sigma-Aldrich Co. (Sigma-Aldrich Chemie GmbH Export Department, Eschenstrasse 5, 82024 Taufkirchen, Germany). *E. coli* strains DH5α and BL21(DE3)pLysS were purchased from Tiangen (Tiangen Biotech, Beijing, China). All other chemicals were of analytical grade and were purchased from GE Healthcare Bio-Sciences (P.O. Box 643065, Pittsburgh, PA 15264-3065, USA).

### Cell culture and hypoxic treatment

U87MG and HepG2 cells were cultured in Dulbecco’s modified Eagle’s medium (DMEM) supplemented with 10% fetal bovine serum and 1% penicillin-streptomycin at 37 °C in a humidified incubator. For hypoxic treatment, U87MG cells were incubated in a hypoxic chamber containing 1% O_2_, 5% CO_2_, and 94% N_2_ for 6, 24, or 48 h, while control cells were maintained under normoxic conditions.

### Metabolite analysis by mass spectrometry

Cells were harvested and intracellular metabolites were extracted using a precooled extraction solvent. The levels of glucose, sorbitol, and fructose were analyzed by hydrophilic interaction liquid chromatography-tandem mass spectrometry (HILIC-LC-MS/MS) on a 5500 QTRAP mass spectrometer (AB SCIEX, Framingham, MA, USA).

### Construction of SDH expression plasmids

PCR was performed to amplify the sorbitol dehydrogenase (SDH) coding sequence using primers containing EcoRI and XhoI restriction sites (forward primer: 5′-CGGAATTCGCAGCTCCAGCTAAGGGCGAG-3′; reverse primer: 5′-CCCTCGAGCTAGGGGTTTGGTCATTGG-3′). The PCR product and the pET28a vector were digested with EcoRI and XhoI, ligated using T4 DNA ligase, and transformed into DH5α competent cells. Recombinant *E. coli* DH5α cells were cultured in Luria-Bertani (LB) medium supplemented with kanamycin (50 μg/mL). Positive clones were screened, and the recombinant plasmid was extracted using a Plasmid Miniprep Kit. The verified plasmid was then transformed into BL21(DE3)pLysS cells for protein expression (15).

### Construction of LDHA expression plasmids

PCR was performed to amplify the LDHA coding sequence using primers containing HindIII and NotI restriction sites (forward primer: 5′-CCAAGCTTGCGGTGAACCCTCAGGAGGCTAT-3′; reverse primer: 5′-ATTTGCGGCCGCTTAAAATTGCAGCTCCTTTTGG-3′). The PCR product and the pET28a vector were digested with HindIII and NotI, ligated using T4 DNA ligase, and transformed into DH5α competent cells. Recombinant *E. coli* DH5α cells were cultured in LB medium supplemented with kanamycin (50 μg/mL). Positive clones were identified, and the recombinant plasmid was extracted using a Plasmid Miniprep Kit. The verified plasmid was then transformed into BL21(DE3)pLysS cells for protein expression.

### Western blotting

Cells were lysed in RIPA buffer (Solarbio, Beijing, China) supplemented with Halt protease/phosphatase inhibitor cocktail (Pierce Biotechnology, Rockford, USA) on ice for 30 min, followed by centrifugation at 12,000 × g for 15 min at 4 °C. Protein concentrations were determined using a BCA kit (Beyotime, Shanghai, China). Lysates (150-250 μg) were separated by SDS-PAGE (Seven Easy PAGE, Seven/Abcells, Beijing, China), transferred to PVDF membranes, and probed with the following antibodies: SDH (1:1000, EPR15857), LDHA (1:1000, ab191332), all from Abcam.

### Cloning, expression, purification, and identification of SDH and LDHA

A single confirmed colony was inoculated into LB medium containing kanamycin (50 μg/mL) and cultured at 37 °C. When the bacterial density reached an OD600 of 0.3-0.5, protein expression was induced with 0.1 mM isopropyl β-D-1-thiogalactopyranoside (IPTG), followed by incubation at 18 °C for 24 h. BL21(DE3)pLysS host cells without plasmid were used as the negative control. Recombinant protein expression was examined by Western blotting. Cells were harvested by centrifugation at 7000 × g for 10 min and washed twice with cold phosphate-buffered saline (PBS). The cell pellets were resuspended in lysis buffer and disrupted by sonication on ice. The lysates were then centrifuged at 20000 × g at 4°C for 20 min to remove cell debris (16, 17). The supernatants were applied to a Ni-NTA affinity column for purification of His-tagged proteins. Bound proteins were eluted stepwise with imidazole-containing buffer (50 mmol/L NaH_2_PO_4_, 300 mmol/L NaCl, and 50, 100, 150, 200, 250, or 500 mmol/L imidazole, pH 8.0). Eluted fractions were analyzed by SDS-PAGE, and the identity of recombinant proteins was further confirmed by Western blotting. Protein concentrations were determined using the Bradford method by measuring absorbance at 595 nm (18).

### Enzyme kinetic assays

The kinetic properties of recombinant SDH and LDHA were determined in a 300 μL reaction mixture containing PBS buffer (pH 8.0) at 25°C or 37°C. For the sorbitol oxidation reaction, the standard reaction mixture contained 100 mM sorbitol and 0.25 mM NAD^+^. For the fructose reduction reaction, the standard reaction mixture contained 100 mM fructose and 0.25 mM NADH. To determine kinetic parameters, 400 ng of LDHA or SDH was added to each reaction, and either sorbitol/fructose (100 to 3.125 mM, two-fold serial dilution) or NAD^+^/NADH (0.5 to 0.03 mM, two-fold serial dilution) was used as the variable substrate while the co-substrate concentration was kept constant. Reactions were monitored for 1 min by measuring the change in absorbance at 340 nm. Heat-inactivated LDHA or SDH was used as a negative control. *K*m and *V*max values were calculated from Lineweaver-Burk plots, and *k*cat was calculated according to the equation *k*cat = *V*max/*E*.

### LDHA knockdown and overexpression

For LDHA knockdown, U87MG cells were transfected with small interfering RNA targeting LDHA (siLDHA) or a negative control siRNA (siCtrl) using a transfection reagent according to the manufacturer’s instructions. The siLDHA sequence used was 5′-GGTACCACTTCCATTGTAAGT-3′, and the negative control siRNA sequence was 5′-CCTAAGGTTAAGTCGCCCTCG-3′. For LDHA overexpression, U87MG cells were transiently transfected with a pcDNA3.1-LDHA plasmid containing the full-length human LDHA coding sequence. After transfection, cells were cultured for 24–48 h to allow gene silencing or overexpression, and then exposed to hypoxic conditions for 24 h. Cells were subsequently collected for Western blotting, intracellular metabolite analysis, ROS detection, and cell viability assays. The efficiency of LDHA knockdown or overexpression was confirmed by Western blotting.

### Intracellular ROS detection

Intracellular ROS levels were measured using a DCFH-DA-based ROS detection assay. Briefly, after LDHA knockdown or overexpression followed by hypoxic treatment for 24 h, U87MG cells were washed with PBS and incubated with DCFH-DA working solution at 37 °C in the dark according to the manufacturer’s instructions. After incubation, the cells were washed to remove excess probe, and fluorescence intensity was measured using a fluorescence microplate reader. The relative ROS level was calculated based on fluorescence intensity and normalized to the hypoxic control group.

### Cell viability assay

Cell viability was assessed using a CCK-8 assay according to the manufacturer’s instructions. Briefly, U87MG cells were seeded into 96-well plates and subjected to LDHA knockdown or overexpression followed by hypoxic treatment for 24 h. CCK-8 reagent was added to each well and incubated at 37°C. Absorbance was measured at 450 nm using a microplate reader. Cell viability was expressed relative to the hypoxic control group.

### Statistical analysis

All experiments were performed in at least triplicate unless otherwise indicated. Data are presented as mean ± SD. Comparisons between two groups were performed using unpaired two-tailed Student’s t-test. Comparisons among multiple groups were performed using one-way ANOVA test. P < 0.05 was considered statistically significant.

## Results

### Hypoxia promotes sorbitol-to-fructose metabolism in U87MG tumor cells

**Figure 1:**
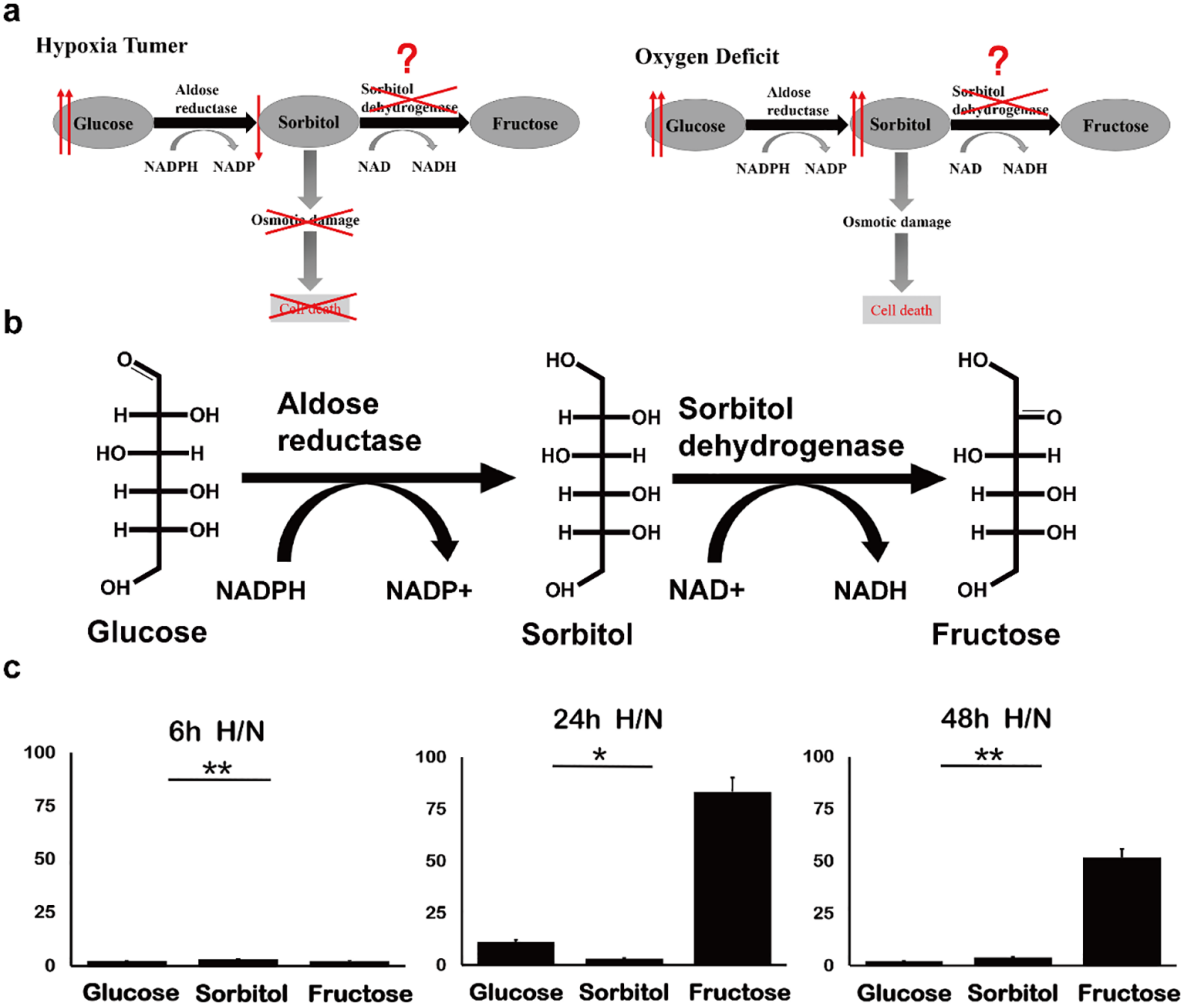
Hypoxia promotes sorbitol-to-fructose metabolism in U87MG tumor cells. **(a)** Schematic illustration of the polyol pathway in tumor cells under hypoxic conditions. Glucose is reduced to sorbitol by aldose reductase using NADPH, and sorbitol is subsequently oxidized to fructose in an NAD^+^ dependent manner. **(b)** Chemical reaction scheme showing the conversion of glucose to sorbitol and sorbitol to fructose through the polyol pathway. **(c)** Mass spectrometric analysis of intracellular glucose, sorbitol, and fructose in U87MG cells cultured under hypoxic and normoxic conditions for 6, 24, and 48 h. Bars represent the hypoxia/normoxia (H/N) ratios of the indicated metabolites. Data are shown as mean ± SD (n=3). *P < 0.05, **P < 0.01.

To examine whether the polyol pathway remains active in tumor cells, U87MG cells were cultured under normoxic or hypoxic conditions, and intracellular metabolites were analyzed by mass spectrometry. As shown in **Figures 1a, b**, glucose can be converted to sorbitol by aldose reductase and subsequently oxidized to fructose in an NAD^+^ dependent manner. Under hypoxic conditions, the hypoxia/normoxia ratios of sorbitol were 3.13, 3.07, and 3.83 at 6, 24, and 48 h, respectively, whereas the corresponding ratios of fructose were 2.17, 83.47, and 51.75. As shown in **Figure 1c**, fructose accumulation became particularly prominent at 24 and 48 h, indicating that sorbitol was actively metabolized to fructose in hypoxic tumor cells. Considering that sorbitol dehydrogenase is expressed at low levels in some tumor cells, these results suggest that another enzyme with a similar catalytic function may participate in this metabolic process.

### Recombinant SDH and LDHA were successfully expressed and purified

**Figure 2:**
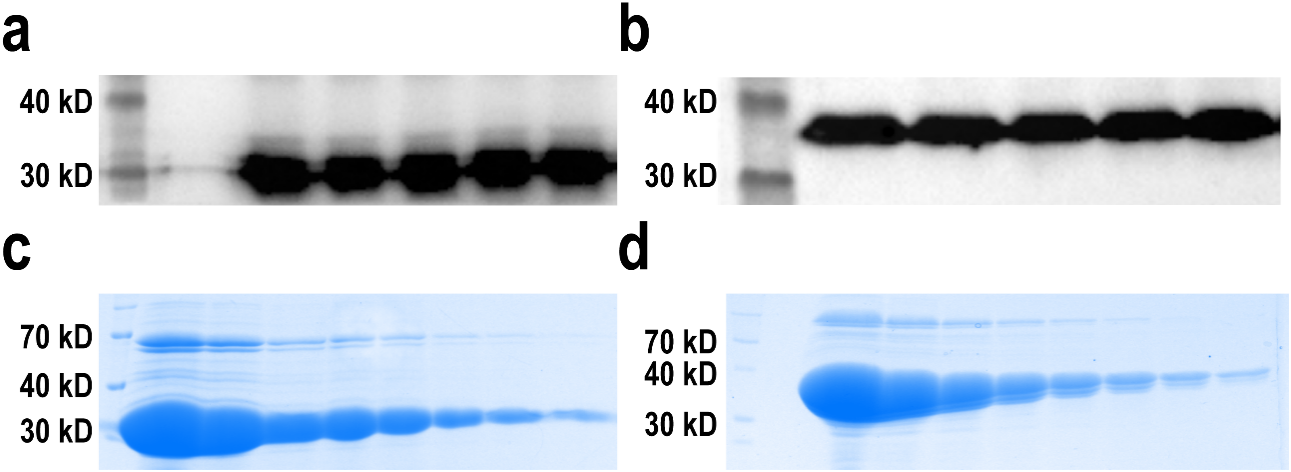
Expression and purification of recombinant SDH and LDHA. **(a)** Western blot analysis of recombinant SDH expressed in *E. coli*, showing a band at approximately 30 kDa. **(b)** Western blot analysis of recombinant LDHA expressed in *E. coli*, showing a band at approximately 39 kDa. **(c)** SDS-PAGE analysis of purified SDH after Ni-Sepharose affinity chromatography. **(d)** SDS-PAGE analysis of purified LDHA after Ni-Sepharose affinity chromatography.

To compare the catalytic properties of SDH and LDHA, recombinant proteins were expressed in E. coli and purified by Ni-Sepharose affinity chromatography. Western blot analysis confirmed that SDH and LDHA were successfully expressed at the expected molecular weights of approximately 30 kDa and 39 kDa, respectively (**Figures 2a, b**). SDS-PAGE further demonstrated successful purification and enrichment of both proteins (**Figures 2c, d**). After purification and concentration (19, 20), the final protein concentrations reached 0.58 mg/mL for SDH and 0.37 mg/mL for LDHA, indicating that both enzymes were obtained at sufficient purity and yield for subsequent kinetic analyses.

### Comparative kinetic analysis of LDHA and SDH in sorbitol/fructose-related reactions

**Figure 3:**
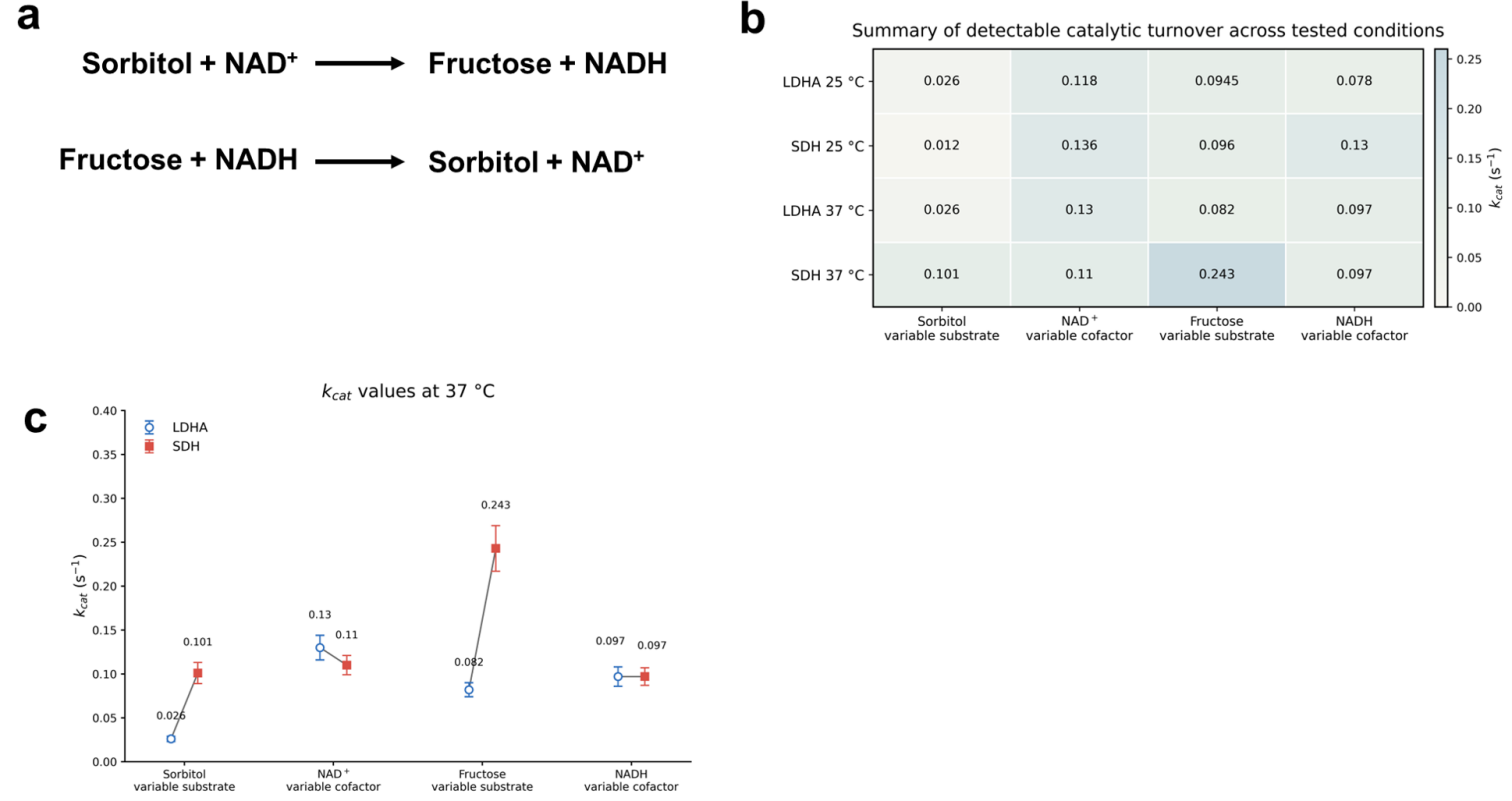
Comparative kinetic analysis of LDHA and SDH in sorbitol/fructose-related reactions. **(a)** Schematic overview of the two reaction directions examined in the enzyme kinetic assays: sorbitol oxidation coupled with NAD^+^ reduction, and fructose reduction coupled with NADH oxidation. **(b)** Heatmap summary of kcat values for LDHA and SDH at 25°C and 37°C under four kinetic conditions, including sorbitol as the variable substrate, NAD^+^ as the variable cofactor, fructose as the variable substrate, and NADH as the variable cofactor. **(c)** Comparison of kcat values for LDHA and SDH at 37°C. Data are shown as mean ± SD from 3 independent enzymatic assays.

To further characterize the sorbitol/fructose-related catalytic activities of LDHA and SDH, enzyme kinetic assays were performed in two reaction directions: sorbitol oxidation coupled with NAD^+^ reduction and fructose reduction coupled with NADH oxidation (21–25). In each reaction direction, either the sugar substrate or the corresponding cofactor was used as the variable component while the other component was kept constant (**Figure 3a**).

The reactions were examined at both 25 °C and 37 °C. The 25 °C condition was included as a commonly used room-temperature condition for *in vitro* enzymatic assays, whereas the 37 °C condition was used to evaluate catalytic activity under a physiologically relevant temperature. To facilitate direct comparison between the two enzymes, the kinetic parameters obtained at 25 °C and 37 °C were summarized in **Tables 1**, **2**, respectively. The corresponding Michaelis-Menten and Lineweaver-Burk analyses are shown in **Supplementary Figures 1**-**4**.

**Table 1:** Kinetic parameters of sorbitol dehydrogenase and lactate dehydrogenase at 25°C.

Reaction direction	Variable substrate/cofactor	Kinetic parameter	LDHA	SDH
Sorbitol+NAD+ 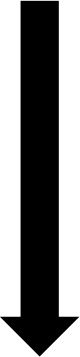 Fructose+NADH	SOR	Vmax (mM/min)	0.010 ± 0.001	0.004 ± 0.001
		Km (mM)	3.745 ± 0.334	2.733 ± 0.234
		Kcat	0.026 ± 0.003	0.012 ± 0.001
	NAD+	Vmax (mM/min)	0.047 ± 0.006	0.054 ± 0.004
		Km (mM)	0.870 ± 0.082	0.820 ± 0.059
		Kcat	0.118 ± 0.010	0.136 ± 0.014
Fructose+NADH 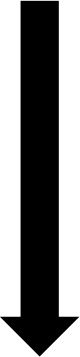 Sorbitol+NAD+	Fru	Vmax (mM/min)	0.037 ± 0.002	0.038 ± 0.003
		Km (mM)	16.870 ± 1.953	21.062 ± 2.282
		Kcat	0.094 ± 0.008	0.096 ± 0.010
	NADH	Vmax (mM/min)	0.031 ± 0.003	0.052 ± 0.003
		Km (mM)	0.173 ± 0.022	0.264 ± 0.031
		Kcat	0.078 ± 0.007	0.130 ± 0.010

**Table 2:** Kinetic parameters of sorbitol dehydrogenase and lactate dehydrogenase at 37°C.

Reaction direction	Variable substrate/cofactor	Kinetic parameter	LDHA	SDH
Sorbitol+NAD+ 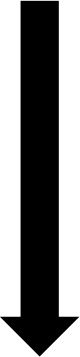 Fructose+NADH	SOR	Vmax (mM/min)	0.010 ± 0.001	0.040 ± 0.004
		Km (mM)	3.100 ± 0.291	0.912 ± 0.083
		Kcat	0.026 ± 0.003	0.101 ± 0.012
	NAD+	Vmax (mM/min)	0.052 ± 0.006	0.040 ± 0.005
		Km (mM)	2.350 ± 0.212	0.996 ± 0.094
		Kcat	0.130 ± 0.014	0.110 ± 0.011
Fructose+NADH 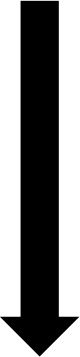 Sorbitol+NAD+	Fru	Vmax (mM/min)	0.033 ± 0.004	0.087 ± 0.009
		Km (mM)	2.373 ± 0.246	1.600 ± 0.171
		Kcat	0.082 ± 0.008	0.243 ± 0.026
	NADH	Vmax (mM/min)	0.039 ± 0.004	0.039 ± 0.004
		Km (mM)	0.095 ± 0.010	0.065 ± 0.007
		Kcat	0.097 ± 0.011	0.097 ± 0.010

As shown in **Figure 3b**, both LDHA and SDH showed detectable catalytic turnover in all tested reactions at 25 °C. The kcat values of LDHA ranged from 0.026 to 0.118 s^-^¹, while those of SDH ranged from 0.012 to 0.136 s^-^¹. These data indicate that both enzymes exhibited measurable sorbitol/fructose-related catalytic activity under the room-temperature *in vitro* assay condition.

The 37 °C data were then used for the main comparison. In the sorbitol oxidation reaction, LDHA showed a kcat value of 0.130 s^-^¹ when NAD^+^ was used as the variable cofactor, which was close to the value observed for SDH under the same condition (0.110 s^-^¹). When sorbitol was used as the variable substrate, the kcat values of LDHA and SDH were 0.026 and 0.101 s^-^¹, respectively.

A similar pattern was observed in the fructose reduction reaction. When NADH was used as the variable cofactor, LDHA and SDH showed the same kcat value of 0.097 s^-^¹ at 37 °C. When fructose was used as the variable substrate, the kcat values of LDHA and SDH were 0.082 and 0.243 s^-^¹, respectively.

Overall, at 37 °C, the kcat values of LDHA and SDH differed by less than one order of magnitude across all tested reactions (**Figure 3c**). When NAD^+^ or NADH was used as the variable cofactor, the two enzymes showed comparable catalytic turnover. When sorbitol or fructose was used as the variable substrate, SDH showed numerically higher kcat values, while LDHA still maintained detectable catalytic activity within the same magnitude range. These results demonstrate that LDHA possesses measurable sorbitol/fructose-related catalytic activity *in vitro* under physiologically relevant temperature.

### LDHA perturbation affects hypoxia-induced sorbitol/fructose metabolism in U87MG tumor cells

**Figure 4:**
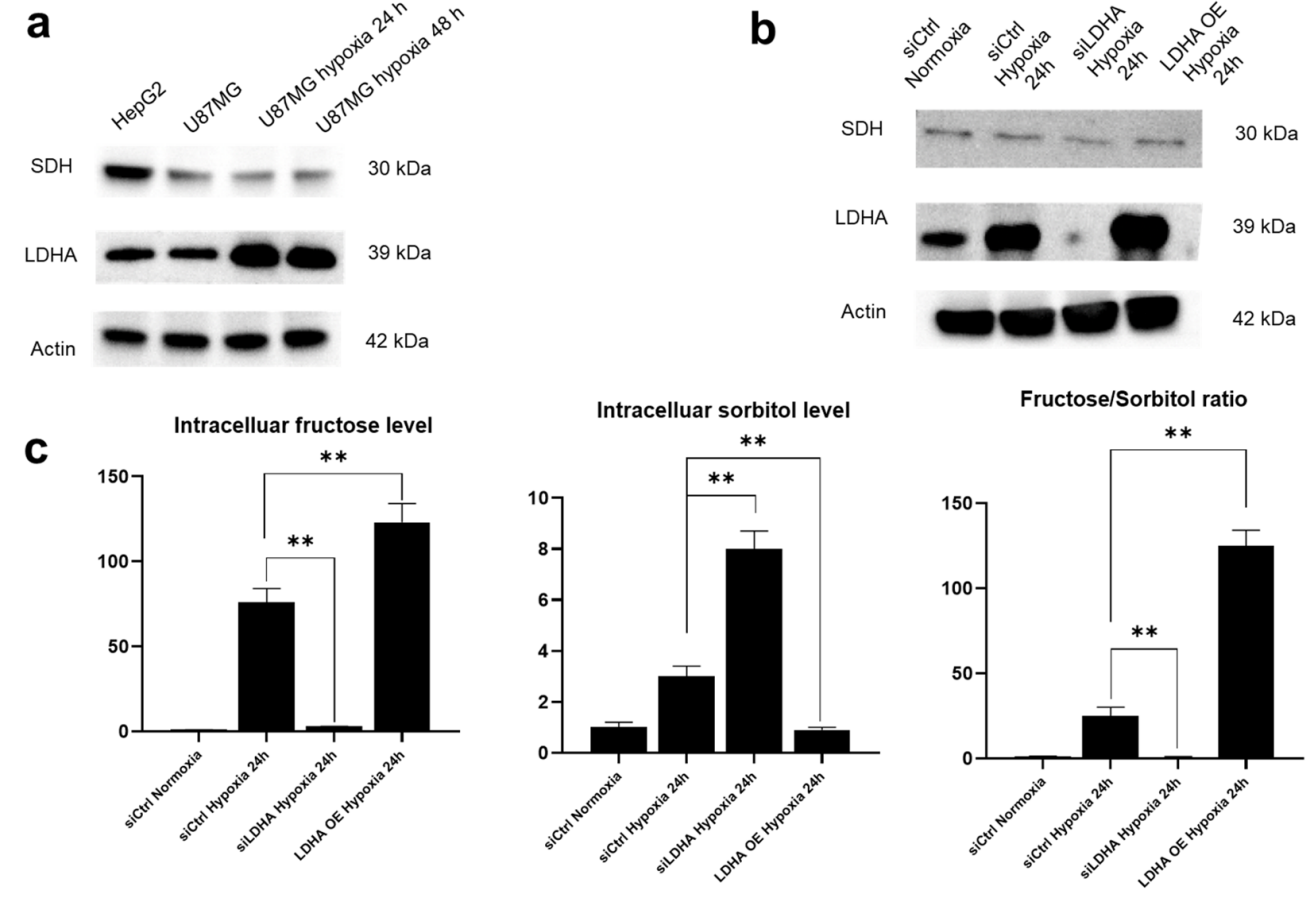
LDHA perturbation affects hypoxia-induced sorbitol/fructose metabolism in U87MG tumor cells. **(a)** Western blot analysis of SDH and LDHA expression in HepG2 cells and U87MG cells under normoxic or hypoxic conditions. U87MG cells were exposed to hypoxia for 24 h or 48 h. Actin was used as the loading control. **(b)** Western blot analysis confirming LDHA knockdown and LDHA overexpression in U87MG cells under hypoxia for 24 h. SDH expression was also examined under the same conditions. Actin was used as the loading control. **(c)** Intracellular fructose level, sorbitol level, and fructose/sorbitol ratio in U87MG cells after LDHA knockdown or LDHA overexpression under hypoxia for 24 h. Metabolite levels were normalized to cell number. Data are shown as mean ± SD from 3 independent experiments. **P < 0.01.

To further evaluate whether LDHA is associated with sorbitol/fructose metabolism in cells, we examined the expression of SDH and LDHA in U87MG cells under normoxic and hypoxic conditions. HepG2 cells were included as a liver-derived reference cell line with detectable SDH expression. Compared with HepG2 cells, U87MG cells showed relatively low SDH protein expression, whereas LDHA was readily detectable. Under hypoxic treatment, LDHA expression increased in U87MG cells at 24 h and 48 h, while SDH remained at a relatively low level (**Figure 4a**).

We next perturbed LDHA expression in U87MG cells and measured intracellular sorbitol and fructose levels after 24 h of hypoxia. Western blot analysis confirmed efficient LDHA knockdown in siLDHA-transfected cells and increased LDHA expression in LDHA-overexpressing cells. SDH expression showed no obvious parallel change among these groups (**Figure 4b**).

Under hypoxia, LDHA knockdown reduced intracellular fructose levels, whereas LDHA overexpression increased fructose accumulation (**Figure 4c**). In contrast, intracellular sorbitol levels increased after LDHA knockdown and decreased after LDHA overexpression. Consistently, the fructose/sorbitol ratio was reduced by LDHA knockdown and increased by LDHA overexpression under hypoxic conditions.

Together, these results indicate that LDHA perturbation affects the balance between sorbitol and fructose in hypoxic U87MG cells. Combined with the *in vitro* kinetic data, these cellular findings support a contribution of LDHA to hypoxia-associated sorbitol/fructose metabolism in a cellular context characterized by relatively low SDH expression.

### LDHA perturbation affects redox balance and cell viability in hypoxic U87MG cells

**Figure 5:**
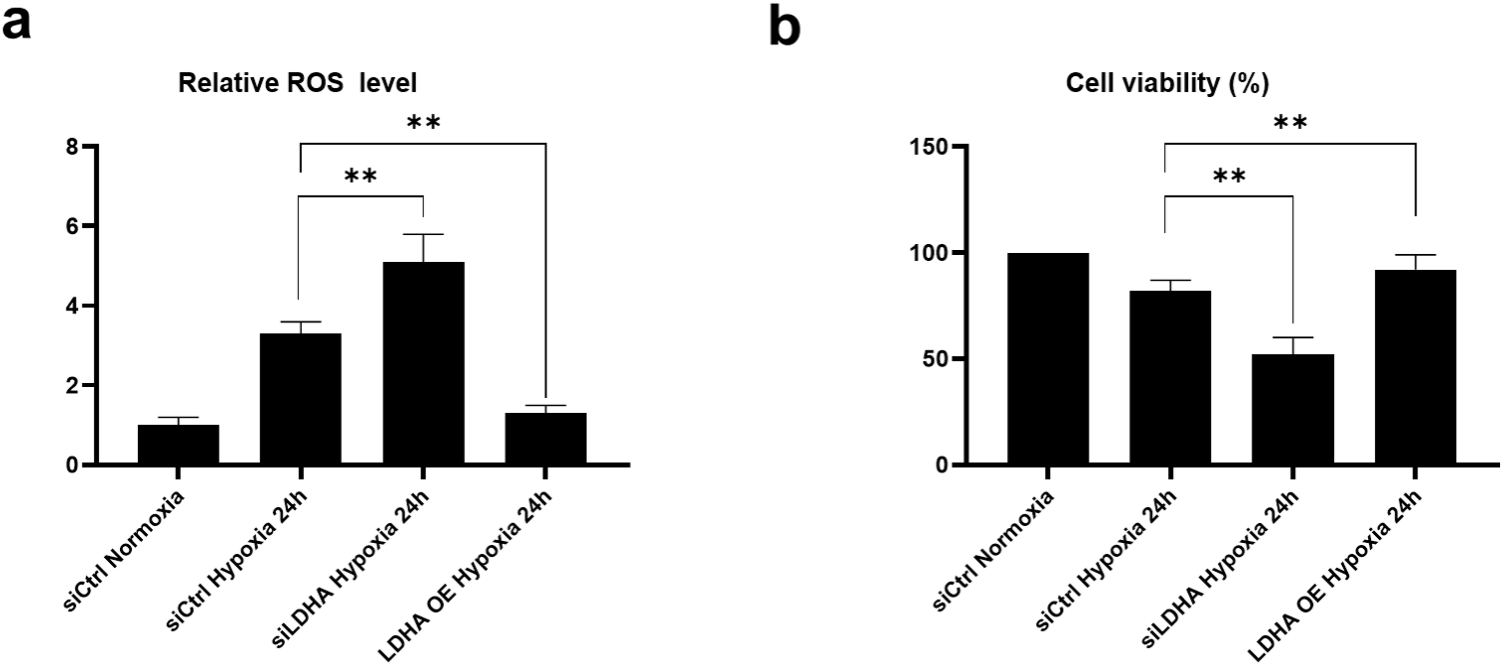
LDHA perturbation affects redox balance and cell viability in hypoxic U87MG cells. **(a)** Relative intracellular ROS levels in U87MG cells after LDHA knockdown or LDHA overexpression under hypoxia for 24 h. **(b)** Cell viability of U87MG cells after LDHA knockdown or LDHA overexpression under hypoxia for 24 h. Data are shown as mean ± SD from 3 independent experiments. **P < 0.01.

To further examine whether LDHA perturbation was associated with hypoxia-related cellular phenotypes, we measured intracellular ROS levels and cell viability in U87MG cells after LDHA knockdown or overexpression under hypoxic conditions. Compared with hypoxic control cells, LDHA knockdown increased intracellular ROS levels, whereas LDHA overexpression reduced ROS accumulation under hypoxia (**Figure 5a**). These results indicate that LDHA perturbation affects the redox status of hypoxic U87MG cells.

We next assessed cell viability under the same conditions. LDHA knockdown decreased cell viability after 24 h of hypoxia, while LDHA overexpression partially restored cell viability compared with LDHA knockdown cells (**Figure 5b**). Together, these results indicate that LDHA is associated not only with hypoxia-related sorbitol/fructose metabolic changes, but also with redox balance and cell viability in U87MG cells under hypoxic stress.

## Discussion

After more than half a century of rapid development, cancer research has generated a rich and complex body of knowledge, revealing that cancer is a disease involving dynamic genomic and metabolic alterations (26). However, many aspects of tumor metabolic reprogramming remain unclear. One of the best-known metabolic features of tumor cells is their high glycolytic activity, which may also increase carbon flux through alternative glucose metabolic branches, including the polyol pathway (27). The polyol pathway is a two-step metabolic process in which glucose is reduced to sorbitol and sorbitol is subsequently oxidized to fructose. This pathway has been widely studied in the context of diabetic hyperglycemia, where it becomes activated under conditions of elevated glucose availability (9). Excessive sorbitol accumulation can increase intracellular osmotic stress and contribute to the development of diabetic complications such as cataracts, neuropathy, and nephropathy (28).

In the present study, our metabolomic data showed that fructose accumulated markedly in U87MG cells under hypoxic conditions, particularly after 24 and 48 h of treatment. This finding suggests that the conversion of sorbitol to fructose remains active in tumor cells even under metabolic stress. Because SDH is the classical enzyme responsible for this step in the polyol pathway, these results initially raised the question of how this conversion is maintained in tumor cells in which SDH expression is limited. Previous studies have shown that SDH is expressed at relatively high levels in some tumors, including cancers of the digestive and endocrine systems, whereas its expression is low in other tumor cell types (29). Based on this background, we hypothesized that another enzyme with related catalytic potential might contribute to sorbitol metabolism in tumor cells.

To test this possibility, recombinant SDH and LDHA were expressed, purified, and subjected to comparative kinetic analysis. The assays were performed under four conditions covering both sorbitol oxidation and fructose reduction. Although 25 °C was included as a commonly used room-temperature condition for *in vitro* enzymatic assays, the physiological interpretation was mainly based on the 37 °C data. At 37 °C, LDHA showed measurable catalytic turnover in all tested sorbitol/fructose-related reactions. When NAD^+^ or NADH was used as the variable cofactor, LDHA showed catalytic turnover comparable to that of SDH. When sorbitol or fructose was used as the variable substrate, SDH showed higher kcat values than LDHA, indicating that SDH remains the more efficient enzyme for the classical polyol-pathway reaction. Nevertheless, the kcat values of LDHA and SDH differed by less than one order of magnitude across the tested 37 °C conditions. These kinetic comparisons were intended to evaluate LDHA and SDH side by side under the same sorbitol/fructose-related reaction conditions, rather than to compare LDHA’s noncanonical sorbitol-related activity with its canonical pyruvate/lactate activity.

It should be noted that the sorbitol/fructose-related activity of LDHA represents a noncanonical catalytic activity and is therefore less efficient than its canonical pyruvate/lactate reaction. In addition, the Km value for sorbitol obtained *in vitro* is higher than the estimated bulk intracellular sorbitol concentration. These observations suggest that LDHA is unlikely to act as a general substitute for SDH in the classical polyol pathway. However, they do not exclude a context-dependent contributory role of LDHA under hypoxic conditions. First, LDHA expression was increased during hypoxia, which may increase the total cellular capacity for low-level sorbitol/fructose-related turnover. Second, the metabolic changes were assessed after 24–48 h of hypoxic exposure, allowing a small but sustained flux to accumulate over time rather than being reflected by instantaneous catalytic efficiency alone. Third, because SDH expression remained relatively low in U87MG cells, even a modest LDHA-associated flux could become detectable in the intracellular sorbitol/fructose balance. Finally, local substrate and cofactor availability within specific intracellular microenvironments may differ from estimated bulk cellular concentrations, potentially permitting low-level LDHA-dependent turnover despite a relatively high apparent Km. Therefore, our findings support a sustained, context-dependent contribution of LDHA to hypoxia-associated sorbitol/fructose metabolism rather than a direct replacement of SDH.

These findings are important because they suggest a possible metabolic link between the glycolytic machinery and the polyol pathway in tumor cells. LDHA is a key glycolytic enzyme that is highly relevant to the metabolic phenotype of cancer cells (30–32). Beyond the biochemical assays, the cellular perturbation experiments further supported the involvement of LDHA in hypoxia-associated sorbitol/fructose metabolism. In U87MG cells with relatively low SDH expression, LDHA knockdown reduced fructose accumulation and increased sorbitol levels under hypoxia, whereas LDHA overexpression produced the opposite pattern. These results indicate that LDHA-associated changes are reflected not only in recombinant enzyme activity but also in intracellular sorbitol/fructose balance. Together with the observation that LDHA is further upregulated under hypoxic conditions, these findings support that LDHA contributes to hypoxia-associated sorbitol/fructose metabolism in a low-SDH cellular context. Such a mechanism may help explain why fructose accumulation can still be detected in hypoxic tumor cells despite the reported low expression of SDH in certain cancers. Although the present data provide biochemical and cellular evidence that LDHA contributes to hypoxia-associated sorbitol/fructose metabolic balance, isotope-tracing flux analysis and *in vivo* validation will be valuable in future studies to further determine the quantitative contribution and physiological relevance of LDHA-dependent carbon flow through this pathway.

It is well known that enzymatic activity plays an important role in the diagnosis and treatment of many diseases. Enzymes may serve not only as biomarkers but also as therapeutic targets. Accordingly, many treatment strategies are based on activation or inhibition of disease-associated enzymes. Numerous chemicals and drugs can influence enzyme activity, and several enzymes, including carbonic anhydrase, paraoxonase, and SDH, have been investigated as pharmacological targets. Previous studies have shown that inhibitors of sorbitol dehydrogenase may help prevent pathological damage associated with excessive polyol-pathway activity, particularly in diabetic complications such as neuropathy, retinopathy, and angiopathy (33). Our findings extend this concept by showing that LDHA, beyond its established role in glycolysis, may also participate in sorbitol/fructose-related metabolism in hypoxic U87MG cells. In addition, LDHA knockdown increased ROS accumulation and reduced cell viability under hypoxia, whereas LDHA overexpression showed the opposite trend. These results link LDHA-associated sorbitol/fructose metabolic changes to redox balance and cell viability under hypoxic stress. Therefore, a better understanding of the catalytic and functional relationship between SDH and LDHA may provide new insight into tumor metabolic adaptation and may also support further investigation of enzyme-targeted metabolic strategies in cancer.

Overall, this study demonstrates that LDHA possesses measurable catalytic activity toward sorbitol- and fructose-related reactions and contribute to polyol-pathway-associated metabolism in tumor cells with low SDH expression. Further studies are needed to determine whether this activity is functionally significant *in vivo*, how it is regulated under hypoxia and other tumor-relevant stresses, and whether targeting this metabolic flexibility may have therapeutic value in cancer.

## Data Availability

The raw data supporting the conclusions of this article will be made available by the authors, without undue reservation.
