# Supplementary material for: Lactate dehydrogenase A exhibits sorbitol/fructose-related catalytic activity and contributes to polyol pathway metabolism in tumor cells
Source: Front Endocrinol (Lausanne). 2026 Jul 20;17:1848426. doi: 10.3389/fendo.2026.1848426 (PMC13429448; doi:10.3389/fendo.2026.1848426)
Supplement: Supplementary file 1 [file DataSheet1.pdf]

Lactate Dehydrogenase A Exhibits Sorbitol/Fructose-Related Catalytic Activity and Contributes to Polyol Pathway Metabolism in Tumor Cells

Siting Feng[1], Zhiyuan Hu[2], Hanbing Tong[1] and Hong Zhang[2, *]

[1] First Affiliated Hospital, Dalian Medical University, Dalian, Liaoning (China), 116024.

[2] Central Hospital of Dalian University of Technology, Faculty of Medicine, Dalian University of Technology, Dalian, Liaoning (China), 116024.

[*]Correspondence to: Hong Zhang, Email: zhyks2008@dlut.edu.cn

Table of contents

Supplementary Figures …... S-2

Figure S1. .... S-2

Figure S2. .... S-3

Figure S3. .... S-4

Figure S4. ....S-5

Supplementary Figures

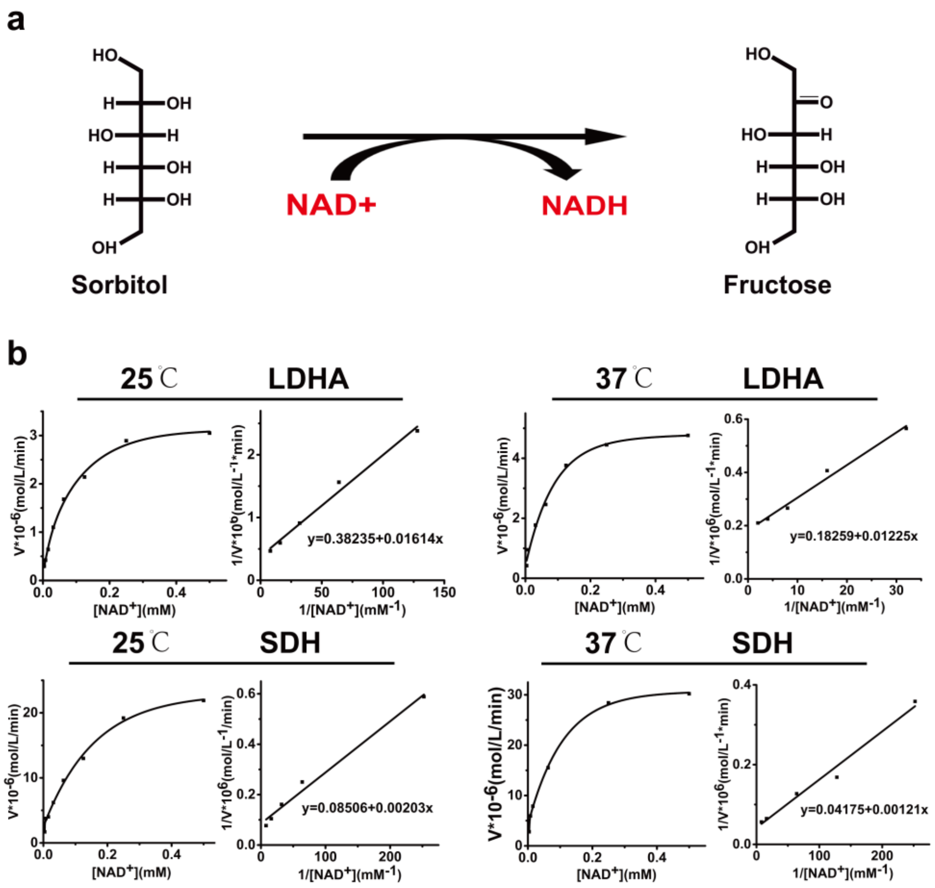

Supplemental Fig. 1. Kinetic analysis of SDH and LDHA in the sorbitol oxidation reaction with NAD^+ as the variable substrate.

(a) Schematic representation of the oxidation of sorbitol to fructose coupled with the reduction of NAD^+ to NADH. (b) Enzyme kinetic analysis of LDHA and SDH at 25 °C and 37 °C with NAD^+ as the variable substrate and sorbitol kept constant. For each enzyme, Michaelis-Menten plots are shown on the left and Lineweaver-Burk double-reciprocal plots are shown on the right. Upper panels, LDHA; lower panels, SDH.

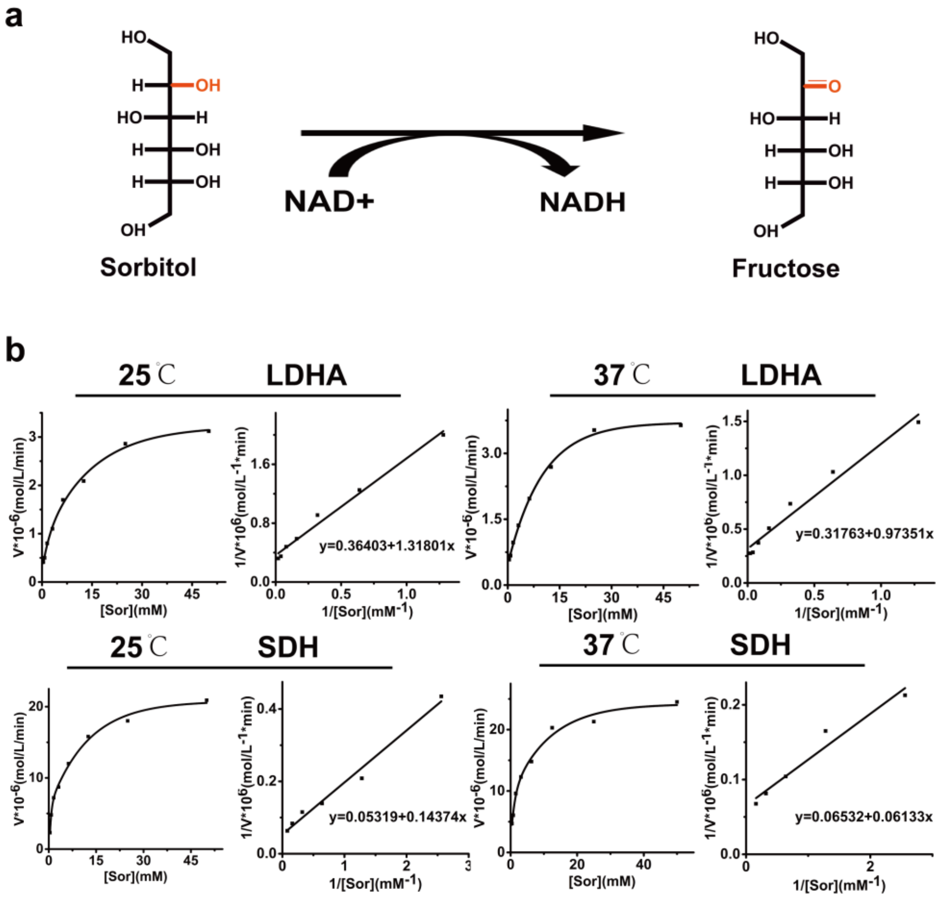

Supplemental Fig. 2. Kinetic analysis of SDH and LDHA in the sorbitol oxidation reaction with sorbitol as the variable substrate.

(a) Schematic representation of the oxidation of sorbitol to fructose coupled with the reduction of NAD^+ to NADH. (b) Enzyme kinetic analysis of LDHA and SDH at 25 °C and 37 °C with sorbitol as the variable substrate and NAD^+ kept constant. For each enzyme, Michaelis-Menten plots are shown on the left and Lineweaver-Burk double-reciprocal plots are shown on the right. Upper panels, LDHA; lower panels, SDH.

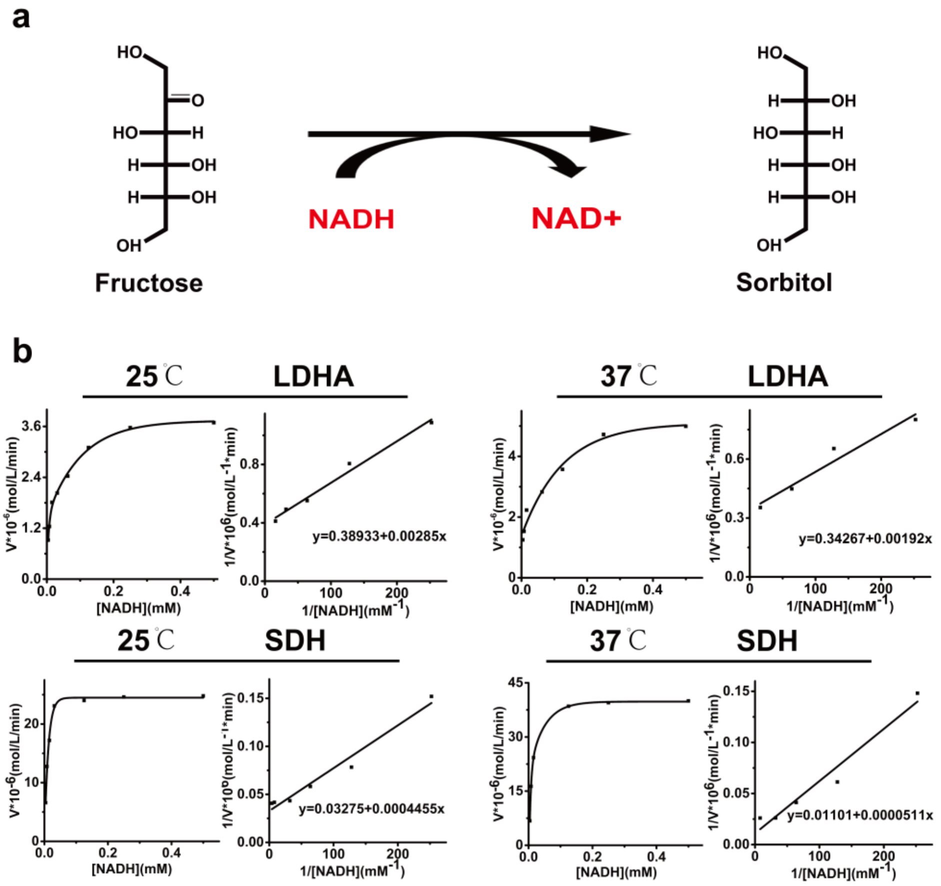

Supplemental Fig. 3. Kinetic analysis of SDH and LDHA in the fructose reduction reaction with NADH as the variable substrate.

(a) Schematic representation of the reduction of fructose to sorbitol coupled with the oxidation of NADH to NAD^+. (b) Enzyme kinetic analysis of LDHA and SDH at 25 °C and 37 °C with NADH as the variable substrate and fructose kept constant. For each enzyme, Michaelis-Menten plots are shown on the left and Lineweaver-Burk double-reciprocal plots are shown on the right. Upper panels, LDHA; lower panels, SDH.

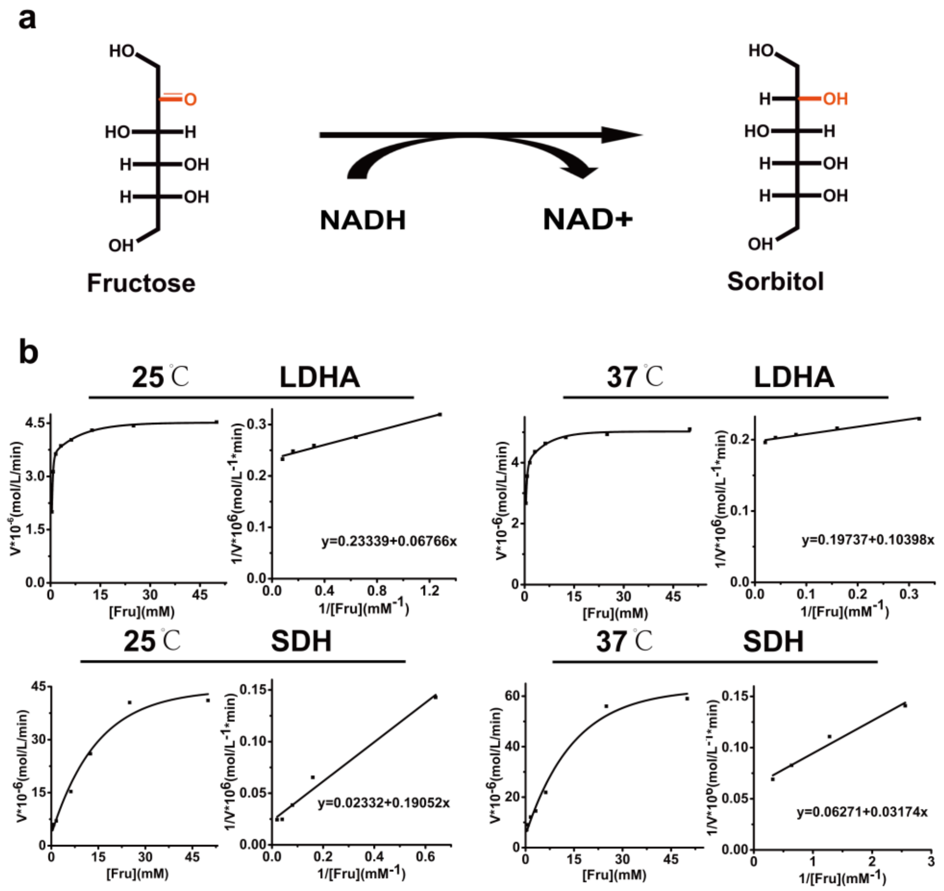

Supplemental Fig. 4. Kinetic analysis of SDH and LDHA in the fructose reduction reaction with fructose as the variable substrate.

(a) Schematic representation of the reduction of fructose to sorbitol coupled with the oxidation of NADH to NAD^+. (b) Enzyme kinetic analysis of LDHA and SDH at 25 °C and 37 °C with fructose as the variable substrate and NADH kept constant. For each enzyme, Michaelis-Menten plots are shown on the left and Lineweaver-Burk double-reciprocal plots are shown on the right. Upper panels, LDHA; lower panels, SDH.
